# Poly[diaqua­bis[μ_2_-2,4-(dichloro­phenoxy)­acetato-κ^2^ *O*:*O*′]iron(II)]

**DOI:** 10.1107/S1600536808029279

**Published:** 2008-09-20

**Authors:** Wei-Bo Pan, Xiao-Hong Xu, Xiao-Hui Huang, Rong-Hua Zeng

**Affiliations:** a School of Chemistry and the Environment, South China Normal University, Guangzhou 510006, People’s Republic of China; b Key Laboratory of Electrochemical Technology, of Energy Storage and Power Generation in Guangdong Universities, Guangzhou 510631, People’s Republic of China

## Abstract

In the title compound, [Fe(C_8_H_5_Cl_2_O_3_)_2_(H_2_O)_2_]_*n*_, the Fe^II^ atom is located on an inversion center. It is coordinated by four O atoms from four 2,4-dichloro­phenoxy­acetate ligands and two water mol­ecules, displaying a distorted octa­hedral geometry. The carboxyl­ate groups of the 2,4-dichloro­phenoxy­acetate ligands link the Fe atoms, forming a polymeric layered network in the *bc* plane. Intra­layer O—H⋯O hydrogen bonds enhance the stability of the two-dimensional network.

## Related literature

For background on supra­molecular networks, see: Eddaoudi *et al.* (2001 ▶); Rizk *et al.* (2005 ▶).
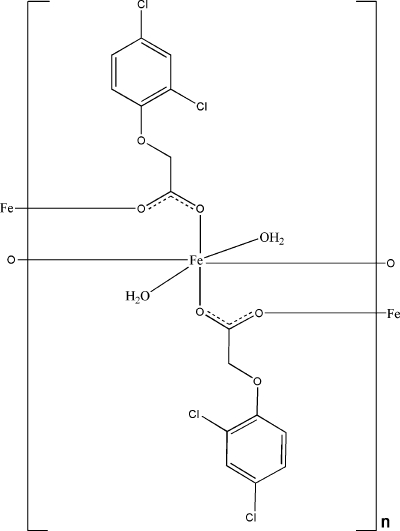


## Experimental

### 

#### Crystal data

[Fe(C_8_H_5_Cl_2_O_3_)_2_(H_2_O)_2_]
*M* *_r_* = 531.92
Monoclinic, 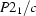

*a* = 17.604 (2) Å
*b* = 7.3122 (8) Å
*c* = 8.0312 (9) Å
β = 94.258 (2)°
*V* = 1031.0 (2) Å^3^
*Z* = 2
Mo *K*α radiation
μ = 1.29 mm^−1^
*T* = 296 (2) K
0.23 × 0.21 × 0.20 mm

#### Data collection

Bruker SMART APEXII CCD area-detector diffractometer
Absorption correction: multi-scan (*SADABS*; Bruker, 2001 ▶) *T*_min_ = 0.756, *T*_max_ = 0.782
5059 measured reflections
1849 independent reflections
1675 reflections with *I* > 2σ(*I*)
*R*_int_ = 0.021

#### Refinement

*R*[*F* ^2^ > 2σ(*F* ^2^)] = 0.034
*wR*(*F* ^2^) = 0.097
*S* = 1.05
1849 reflections
137 parameters
H-atom parameters constrained
Δρ_max_ = 0.48 e Å^−3^
Δρ_min_ = −0.48 e Å^−3^

### 

Data collection: *APEX2* (Bruker, 2007 ▶); cell refinement: *SAINT* (Bruker, 2007 ▶); data reduction: *SAINT*; program(s) used to solve structure: *SHELXS97* (Sheldrick, 2008 ▶); program(s) used to refine structure: *SHELXL97* (Sheldrick, 2008 ▶); molecular graphics: *SHELXTL* (Sheldrick, 2008 ▶); software used to prepare material for publication: *SHELXL97*.

## Supplementary Material

Crystal structure: contains datablocks global. DOI: 10.1107/S1600536808029279/hy2149sup1.cif

Structure factors: contains datablocks I. DOI: 10.1107/S1600536808029279/hy2149Isup2.hkl

Additional supplementary materials:  crystallographic information; 3D view; checkCIF report

## Figures and Tables

**Table:** 

Fe1—O3^i^	2.1654 (17)
Fe1—O2	2.1697 (16)
Fe1—O1*W*	2.2297 (18)

**Table:** 

O3^i^—Fe1—O2	80.18 (6)
O3^ii^—Fe1—O2	99.82 (6)
O3^i^—Fe1—O1*W*	89.36 (7)
O3^ii^—Fe1—O1*W*	90.64 (7)
O2^iii^—Fe1—O1*W*	91.25 (7)
O2—Fe1—O1*W*	88.75 (7)

Symmetry codes: (i) 
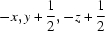
; (ii) 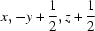; (iii) 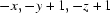.

**Table 2:** Hydrogen-bond geometry (Å, °)

*D*—H⋯*A*	*D*—H	H⋯*A*	*D*⋯*A*	*D*—H⋯*A*
O1*W*—H1*W*⋯O1^iv^	0.82	2.41	3.051 (3)	135
O1*W*—H2*W*⋯O3^iii^	0.82	2.08	2.797 (3)	145

Symmetry codes: (iii) 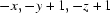; (iv) 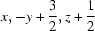.

**Fig. 1.**
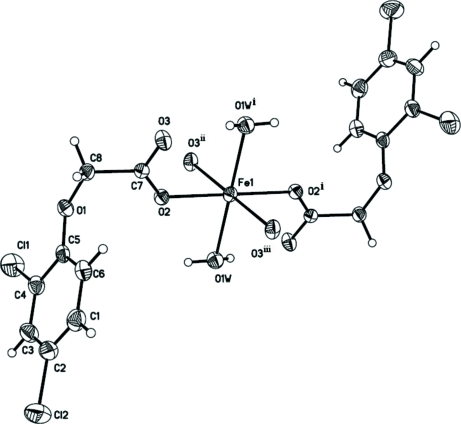
The asymmetric unit of the title compound, together with symmetry-related atoms to complete the coordination units. Displacement ellipsoids are drawn at the 30% probability level. [Symmetry codes: (i)-x, 1-y, 1-z; (ii) x, 1/2-y, 1/2+z; (iii) -x, 1/2+y, 1/2-z.]

**Fig. 2.**
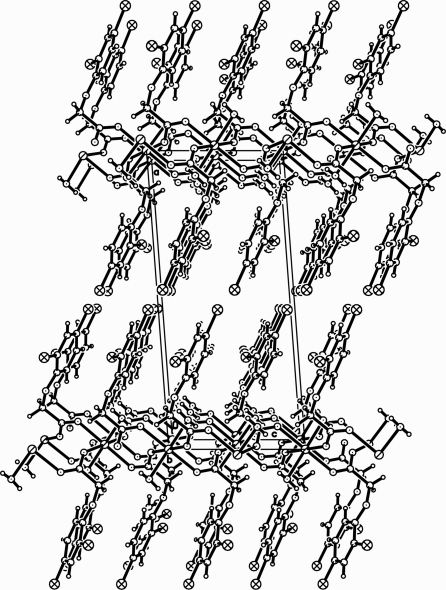
View of the two-dimensional network in the title compound.

## supplementary crystallographic information

### Comment

The design, synthesis, characterization and properties of supramolecular networks formed by using functionalized organic molecules as bridges between metal centers are of great interest (Eddaoudi *et al.*,  2001; Rizk *et al.*,  2005). As a building block, 2,4-dichlorophenoxyacetate is an excellent candidate for the construction of supramolecular complexes. Recently, we obtained the title compound, a new coordination polymer.

In the title compound, the Fe^II^ atom is located on an inversion center and coordinated by four O atoms from four 2,4-dichlorophenoxyacetate ligands and two water molecules in an octahedral geometry (Fig. 1; Table 1). The Fe^II^ atoms are linked by 2,4-dichlorophenoxyacetate ligands to form a polymeric layered network in the *bc*-plane (Fig. 2). The two-dimensional network is further stabilized by intralayer O—H···O hydrogen bonds involving the coordinated water molecules and the O atoms from the ligands (Table 2). The adjacent Fe···Fe separation is 5.431 (4) Å.

### Experimental

A mixture of FeCl_2_ (0.127 g, 1 mmol), 2,4-dichlorophenoxyacetic acid (0.221 g, 1 mmol), NaOH (0.04 g, 1 mmol) and water (10 ml) was stirred vigorously for 20 min, and then sealed in a 20 ml Teflon-lined stainless steel autoclave. The autoclave was heated to and maintained at 433 K for 2 d, and then cooled to room temperature at 5 K h^-1^ to afford red block crystals.

### Refinement

H atoms of water molecule were located in difference Fourier maps and fixed with *U*_iso_(H) = 1.5*U*_eq_(O). C-bound H atoms were positioned geometrically and refined as riding atoms, with C—H = 0.97 (CH_2_) and 0.93 (CH) Å and with *U*_iso_(H) = 1.2*U*_eq_(C).

### Crystal data

**Table:**

[Fe(C_8_H_5_Cl_2_O_3_)_2_(H_2_O)_2_]	*F*(000) = 536
*M**_r_* = 531.92	*D*_x_ = 1.714 Mg m^−^^3^
Monoclinic, *P*2_1_/*c*	Mo *K*α radiation, λ = 0.71073 Å
Hall symbol: -P 2ybc	Cell parameters from 6377 reflections
*a* = 17.604 (2) Å	θ = 1.7–28.0°
*b* = 7.3122 (8) Å	µ = 1.29 mm^−^^1^
*c* = 8.0312 (9) Å	*T* = 296 K
β = 94.258 (2)°	Block, colourless
*V* = 1031.0 (2) Å^3^	0.23 × 0.21 × 0.20 mm
*Z* = 2	

### Data collection

**Table:**

Bruker SMART APEXII CCD area-detector diffractometer	1849 independent reflections
Radiation source: fine-focus sealed tube	1675 reflections with *I* > 2σ(*I*)
graphite	*R*_int_ = 0.021
φ and ω scan	θ_max_ = 25.2°, θ_min_ = 2.3°
Absorption correction: multi-scan (SADABS; Bruker, 2001)	*h* = −21→17
*T*_min_ = 0.756, *T*_max_ = 0.782	*k* = −8→8
5059 measured reflections	*l* = −9→9

### Refinement

**Table:**

Refinement on *F*^2^	Primary atom site location: structure-invariant direct methods
Least-squares matrix: full	Secondary atom site location: difference Fourier map
*R*[*F*^2^ > 2σ(*F*^2^)] = 0.034	Hydrogen site location: inferred from neighbouring sites
*wR*(*F*^2^) = 0.097	H-atom parameters constrained
*S* = 1.05	*w* = 1/[σ^2^(*F*_o_^2^) + (0.0473*P*)^2^ + 1.0193*P*] where *P* = (*F*_o_^2^ + 2*F*_c_^2^)/3
1849 reflections	(Δ/σ)_max_ < 0.001
137 parameters	Δρ_max_ = 0.49 e Å^−^^3^
0 restraints	Δρ_min_ = −0.48 e Å^−^^3^

### Fractional atomic coordinates and isotropic or equivalent isotropic displacement parameters (Å^2^)

**Table:**

	*x*	*y*	*z*	*U*_iso_*/*U*_eq_	
Fe1	0.0000	0.5000	0.5000	0.03073 (17)	
Cl1	0.30460 (6)	0.78166 (12)	0.11801 (14)	0.0735 (3)	
Cl2	0.46770 (6)	0.2467 (2)	0.43433 (14)	0.0928 (4)	
C5	0.24797 (14)	0.4477 (4)	0.1746 (3)	0.0356 (6)	
C4	0.31062 (16)	0.5621 (4)	0.1975 (4)	0.0431 (6)	
C1	0.32151 (18)	0.2103 (5)	0.3170 (4)	0.0530 (8)	
H1	0.3253	0.0913	0.3575	0.064*	
C6	0.25386 (16)	0.2715 (4)	0.2358 (4)	0.0435 (6)	
H6	0.2122	0.1932	0.2226	0.052*	
C2	0.38251 (18)	0.3256 (5)	0.3371 (4)	0.0565 (8)	
C3	0.37794 (18)	0.5028 (5)	0.2792 (4)	0.0553 (8)	
H3	0.4195	0.5812	0.2948	0.066*	
O1	0.18551 (10)	0.5189 (2)	0.0859 (2)	0.0385 (4)	
O2	0.07809 (9)	0.4569 (2)	0.3070 (2)	0.0323 (4)	
C7	0.07058 (13)	0.3655 (3)	0.1753 (3)	0.0277 (5)	
C8	0.12459 (15)	0.3979 (4)	0.0403 (3)	0.0367 (6)	
H8A	0.1456	0.2812	0.0092	0.044*	
H8B	0.0958	0.4467	−0.0575	0.044*	
O3	0.01927 (10)	0.2497 (2)	0.1419 (2)	0.0407 (4)	
O1W	0.09385 (11)	0.6384 (3)	0.6535 (2)	0.0416 (4)	
H1W	0.1227	0.6879	0.5917	0.062*	
H2W	0.0708	0.7130	0.7070	0.062*	

### Atomic displacement parameters (Å^2^)

**Table:**

	*U*^11^	*U*^22^	*U*^33^	*U*^12^	*U*^13^	*U*^23^
Fe1	0.0341 (3)	0.0280 (3)	0.0304 (3)	0.00233 (19)	0.0045 (2)	0.00195 (19)
Cl1	0.0771 (6)	0.0489 (5)	0.0935 (7)	−0.0269 (4)	−0.0007 (5)	0.0116 (4)
Cl2	0.0567 (6)	0.1397 (11)	0.0803 (7)	0.0293 (6)	−0.0068 (5)	0.0080 (7)
C5	0.0338 (13)	0.0400 (13)	0.0344 (13)	−0.0029 (11)	0.0113 (10)	−0.0048 (11)
C4	0.0430 (15)	0.0433 (15)	0.0439 (15)	−0.0095 (12)	0.0089 (12)	−0.0045 (12)
C1	0.0574 (19)	0.0538 (18)	0.0493 (17)	0.0108 (15)	0.0136 (14)	0.0055 (14)
C6	0.0434 (15)	0.0410 (15)	0.0474 (16)	−0.0046 (12)	0.0119 (12)	0.0001 (12)
C2	0.0435 (17)	0.081 (2)	0.0451 (17)	0.0115 (16)	0.0060 (13)	−0.0031 (16)
C3	0.0384 (16)	0.073 (2)	0.0546 (18)	−0.0126 (15)	0.0041 (13)	−0.0088 (16)
O1	0.0344 (10)	0.0370 (10)	0.0444 (10)	−0.0064 (7)	0.0052 (8)	0.0011 (8)
O2	0.0327 (9)	0.0342 (9)	0.0305 (9)	−0.0017 (7)	0.0056 (7)	−0.0074 (7)
C7	0.0291 (12)	0.0234 (11)	0.0305 (12)	0.0049 (9)	0.0025 (9)	0.0012 (9)
C8	0.0369 (13)	0.0429 (15)	0.0306 (12)	−0.0072 (11)	0.0052 (10)	−0.0047 (11)
O3	0.0423 (10)	0.0358 (10)	0.0456 (11)	−0.0128 (8)	0.0140 (8)	−0.0149 (8)
O1W	0.0415 (11)	0.0412 (10)	0.0419 (10)	−0.0042 (9)	0.0030 (8)	−0.0053 (9)

### Geometric parameters (Å, °)

**Table:**

Fe1—O3^i^	2.1654 (17)	C1—H1	0.9300
Fe1—O3^ii^	2.1654 (17)	C6—H6	0.9300
Fe1—O2^iii^	2.1697 (16)	C2—C3	1.377 (5)
Fe1—O2	2.1697 (16)	C3—H3	0.9300
Fe1—O1W	2.2297 (18)	O1—C8	1.417 (3)
Fe1—O1W^iii^	2.2297 (18)	O2—C7	1.250 (3)
Cl1—C4	1.728 (3)	C7—O3	1.253 (3)
Cl2—C2	1.737 (3)	C7—C8	1.513 (3)
C5—O1	1.368 (3)	C8—H8A	0.9700
C5—C6	1.380 (4)	C8—H8B	0.9700
C5—C4	1.386 (4)	O3—Fe1^iv^	2.1654 (17)
C4—C3	1.381 (4)	O1W—H1W	0.8200
C1—C2	1.365 (5)	O1W—H2W	0.8200
C1—C6	1.389 (4)		
			
O3^i^—Fe1—O3^ii^	180.0	C6—C1—H1	120.1
O3^i^—Fe1—O2^iii^	99.82 (6)	C5—C6—C1	120.5 (3)
O3^ii^—Fe1—O2^iii^	80.18 (6)	C5—C6—H6	119.8
O3^i^—Fe1—O2	80.18 (6)	C1—C6—H6	119.8
O3^ii^—Fe1—O2	99.82 (6)	C1—C2—C3	121.0 (3)
O2^iii^—Fe1—O2	180.0	C1—C2—Cl2	119.5 (3)
O3^i^—Fe1—O1W	89.36 (7)	C3—C2—Cl2	119.4 (3)
O3^ii^—Fe1—O1W	90.64 (7)	C2—C3—C4	118.8 (3)
O2^iii^—Fe1—O1W	91.25 (7)	C2—C3—H3	120.6
O2—Fe1—O1W	88.75 (7)	C4—C3—H3	120.6
O3^i^—Fe1—O1W^iii^	90.64 (7)	C5—O1—C8	117.4 (2)
O3^ii^—Fe1—O1W^iii^	89.36 (7)	C7—O2—Fe1	130.51 (15)
O2^iii^—Fe1—O1W^iii^	88.75 (7)	O2—C7—O3	124.9 (2)
O2—Fe1—O1W^iii^	91.25 (7)	O2—C7—C8	119.4 (2)
O1W—Fe1—O1W^iii^	180.00 (7)	O3—C7—C8	115.7 (2)
O1—C5—C6	125.3 (2)	O1—C8—C7	114.6 (2)
O1—C5—C4	116.1 (2)	O1—C8—H8A	108.6
C6—C5—C4	118.6 (3)	C7—C8—H8A	108.6
C3—C4—C5	121.3 (3)	O1—C8—H8B	108.6
C3—C4—Cl1	119.6 (2)	C7—C8—H8B	108.6
C5—C4—Cl1	119.0 (2)	H8A—C8—H8B	107.6
C2—C1—C6	119.7 (3)	C7—O3—Fe1^iv^	139.91 (16)
C2—C1—H1	120.1	H1W—O1W—H2W	112

Symmetry codes: (i) −*x*, *y*+1/2, −*z*+1/2; (ii) *x*, −*y*+1/2, *z*+1/2; (iii) −*x*, −*y*+1, −*z*+1; (iv) −*x*, *y*−1/2, −*z*+1/2.

### Hydrogen-bond geometry (Å, °)

**Table:**

*D*—H···*A*	*D*—H	H···*A*	*D*···*A*	*D*—H···*A*
O1W—H1W···O1^v^	0.82	2.41	3.051 (3)	135
O1W—H2W···O3^iii^	0.82	2.08	2.797 (3)	145

Symmetry codes: (v) *x*, −*y*+3/2, *z*+1/2; (iii) −*x*, −*y*+1, −*z*+1.
